# Prognostic Factors in Anti-Neutrophil Cytoplasmic Antibody-Associated Glomerulonephritis with Severe Glomerular Sclerosis: A National Registry-Based Cohort Study

**DOI:** 10.1155/2018/5653612

**Published:** 2018-06-03

**Authors:** Rune Bjørneklett, Vilde Solbakken, Leif Bostad, Anne-Siri Fismen

**Affiliations:** ^1^Department of Clinical Medicine, University of Bergen, Bergen, Norway; ^2^Emergency Care Clinic, Haukeland University Hospital, Bergen, Norway; ^3^Department of Pathology, Haukeland University Hospital, Bergen, Norway; ^4^Faculty of Health and Social Sciences, Western Norway University of Applied Sciences, Bergen, Norway

## Abstract

**Background:**

Classification of patients with anti-neutrophil cytoplasmic antibody-associated glomerulonephritis (ANCA-GN) into histological classes is useful for predicting a patient's risk of progression to end-stage renal disease (ESRD). However, even in the worst prognostic group, the 5-year end-stage renal disease-free survival rate is as high as 50%.

**Objectives:**

To investigate those prognostic factors indicative of progression to ESRD in patients with ANCA-GN and sclerosing histology.

**Methods:**

Patients from the Norwegian Kidney Biopsy Registry between 1991 and 2012 who had biopsy verified pauci-immune glomerulonephritis, positive ANCA serology, and sclerosing histology were included. Cases with ESRD during follow-up were identified via linkage with the Norwegian Renal Registry. Potential prognostic factors with relevant cut-offs were compared in patients with and without progression to ESRD during follow-up.

**Results:**

Of 23 included patients, 10 progressed to ESRD. ESRD patients had a lower initial estimated glomerular filtration rate (eGFR; 21 versus 52 ml/min/1.73 m^2^) and a lower percentage of normal glomeruli (4% versus 15%). Five-year risks of ESRD with eGFR >15 versus ≤15 ml/min/1.73 m^2^ were 77% and 15%, with percentage normal glomeruli >10% versus ≤10%, 83% and 39%.

**Conclusions:**

eGFR and percentage of normal glomeruli are strong risk factors for ESRD in ANCA-GN with sclerosing histology.

## 1. Introduction

Renal involvement in the form of glomerulonephritis is common and associated with increased morbidity and mortality in patients with anti-neutrophil cytoplasmic antibody- (ANCA-) associated vasculitis (AAV) [1–3]. A clinical presentation involving rapidly progressive glomerulonephritis syndrome with a positive ANCA test is diagnostic for ANCA-associated glomerulonephritis (ANCA-GN), but tissue confirmation by kidney biopsy is preferred whenever feasible [4, 5]. In addition to confirming the diagnosis of ANCA-GN, histological analysis yields important prognostic information [6–13]. In 2010, an international consortium of renal pathologists and nephrologists proposed a histopathological classification schema for ANCA-GN, which consisted of four histological classes: focal (≥50% normal glomeruli), crescentic (≥50% glomeruli with cellular crescents), mixed (<50% normal/crescentic/globally sclerotic glomeruli), and sclerotic (≥50 globally sclerotic glomeruli) [14]. This classification model's capacity to predict end-stage renal disease (ESRD) has now been validated in many different cohorts of patients with ANCA-GN [15–30]. Generally, focal histology is found to be associated with the best outcomes, whereas a sclerotic histology is associated with the poorest prognosis. Meanwhile, the prognostic separation of patients with crescentic versus mixed histology is based on results from various validation studies not particularly useful [16, 31].

Generally, the most clinically valuable prognostic models are those that can be used to guide physicians regarding treatment and follow-up regimens for patients [31, 32]. If left untreated, a patient with ANCA-GN has a very poor prognosis [33]. With adequate treatment, progression to ESRD can be avoided in most patients with focal/crescentic/mixed histology and there are currently no data suggesting that different therapeutic approaches should be used for these groups. In patients with ANCA-GN and a sclerotic histological presentation, one could speculate whether, due to substantial irreversible renal damage, a less intensive/toxic treatment regimen would benefit patients. However, data from several studies shows that an ESRD-free survival rate in patients with sclerotic histology is surprisingly high, being nearly 50% at 5 years [16]. Thus, reducing treatment intensity in cases with a sclerotic histological presentation could deprive many patients from a successful treatment outcome. There are theoretically many causes of the variability in outcomes of patients with ANCA-GN and sclerotic histology. First, the biopsy needle might have hit a fibrotic scar in the kidney that is otherwise not representative of the total renal mass of the particular patient. Second, the formal criterion for sclerotic histology is at least 50% globally sclerotic glomeruli, which allows for 0–49% of normal and/or crescentic glomeruli. Such flexibility in possible histology under this criterion could contribute to the considerable variation in patient outcomes.

Here, using data from the Norwegian Kidney Biopsy Registry (NKBR) and the Norwegian Renal Registry, we have analyzed predictive factors that could be used to stratify patients with ANCA-GN and a sclerotic histological picture based upon risk. Our hypothesis is that such risk stratification is feasible and could increase the clinical utility of histological classification in patients with ANCA-GN.

## 2. Materials and Methods

The Regional Committee for Medical and Health Research Ethics (REC South-East) approved this study.

In the present study, we included patients with ANCA-GN and a sclerotic histological presentation, as previously described [16]. Identification of the study cohort, baseline data, scoring of patients according to the histologic classification model of ANCA-GN, definition of observation period, and end-points have been described in detail earlier and are only briefly reviewed here.

We identified all patients with a biopsy verified ANCA-GN in the NKBR from 1991 to 2012. The criteria for ANCA-GN were the finding of a pauci-immune necrotizing glomerulonephritis and a positive ANCA titer. Baseline data, including sex, age, ANCA serotype, estimated GFR (eGFR), serum-albumin, systolic and diastolic blood-pressure, and proteinuria, were obtained from the NKBR. An experienced renal-pathologist (L.B.) classified all cases according to the histological classification schema of ANCA-GN and those with a sclerotic presentation and at least 10 glomeruli available for analysis were included in the present study cohort. The pathologist also counted for the percentage of glomeruli without global sclerosis or crescents present in each case. The observation period was from time of biopsy to first event, ESRD, death, or the end of the study period 31 December 2012. Further, the observation period was stratified into induction (≤1 year after biopsy) and remission (>1 year after biopsy) phases. The primary end-point of this study was ESRD, defined as the initiation of chronic renal replacement therapy in the form of dialysis or renal transplantation. Cases with ESRD were identified by linkage of the study cohort with the Norwegian Renal Registry. The secondary end-point included death in patients without ESRD, which was identified by linkage of the study cohort with the Population Registry of Norway. We also defined a combined end-point of patients with ESRD or death during follow-up, whichever came first.

We first compared baseline data between patients who survived and those who progressed to ESRD or died during the induction phase. To assess the significance of our findings, the Chi-squared test was used for categorical variables and Mann–Whitney *U* test was used for continuous variables. Second, using Kaplan Meier statistics and log rank tests we analyzed as 1- and 5-year cumulative ESRD-free survival in the whole cohort and after stratification based on baseline eGFR (≤15 ml/>15 ml/min/1.73 m^2^) and baseline percentage glomeruli without global sclerosis or crescents in the biopsy (≤10%/>10%). These analyses were then repeated using the secondary end-point, ESRD, or death. SPSS version 24 was used for statistical analyses.

## 3. Results

**Figure 1:**
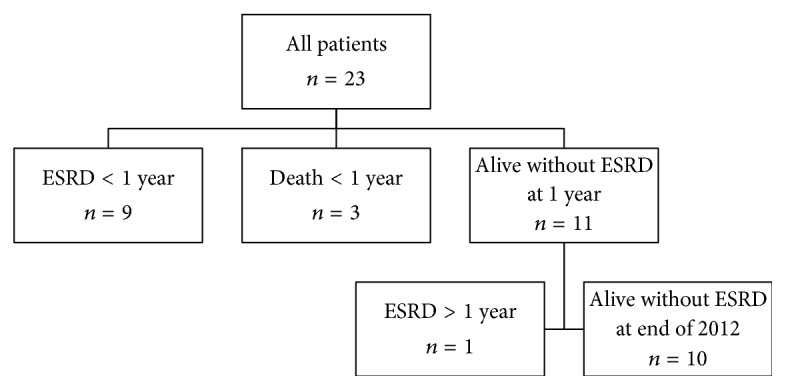
Flow chart demonstrating outcomes in the study cohort. ESRD: end-stage renal disease.

Twenty-three patients with a mean age of 58 years (SD = 21) were included in this study. Of these subjects, 13 (57%) were female. Eighteen (78%) were perinuclear (P) ANCA- or myeloperoxidase (MPO) ANCA-positive and had a mean eGFR of 36 ml/min/1.73 m^2^ (SD = 40) and a mean percentage glomeruli without crescents or global sclerosis of 9% (SD = 12%). Other baseline characteristics are shown in Table 1. The median observation period was 0.7 years (25th–75th percentiles = 0.1–5.3 years) and there were 74 patient years in total. As shown in Figure 1, during the short follow-up period (≤1 year), 9 (39%) patients received chronic renal replacement therapy and 3 (13%) patients without ESRD had died. Eleven (48%) patients survived >1 year after the diagnosis of ANCA-GN without ESRD, 1 (4%) later progressed to ESRD, and 10 (43%) were alive without ESRD by the end of 2012.

A comparison of baseline characteristics between the 11 patients surviving at 1 year after an ANCA-GN diagnosis without ESRD (survivors) and the 12 patients who progressed to ESRD or died is shown in Table 1. The following baseline characteristics differed significantly between survivors and nonsurvivors: eGFR: 52 versus 21 ml/min/1.73 m^2^; serum-albumin: 41 versus 31 g/L; and percentage normal glomeruli: 14 versus 7.

**Figure 2:**
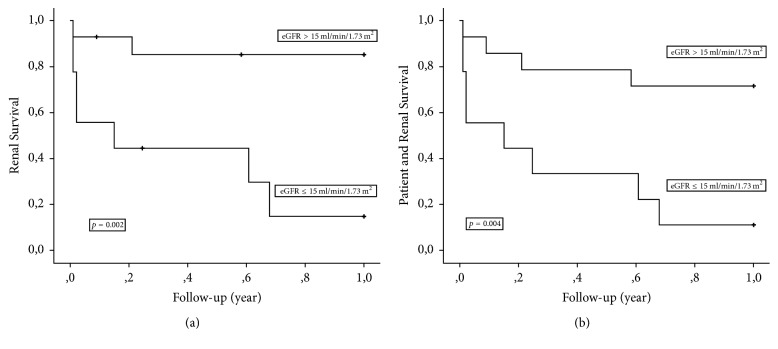
(a) Kaplan Meier plot demonstrating end-stage renal disease-free survival according to baseline estimated glomerular filtration rate ≤ versus >15 ml/min/1.73 m^2^. (b) Kaplan Meier plot demonstrating end-stage renal disease-free patient survival according to baseline estimated glomerular filtration rate ≤ versus >15 ml/min/1.73 m^2^.

**Figure 3:**
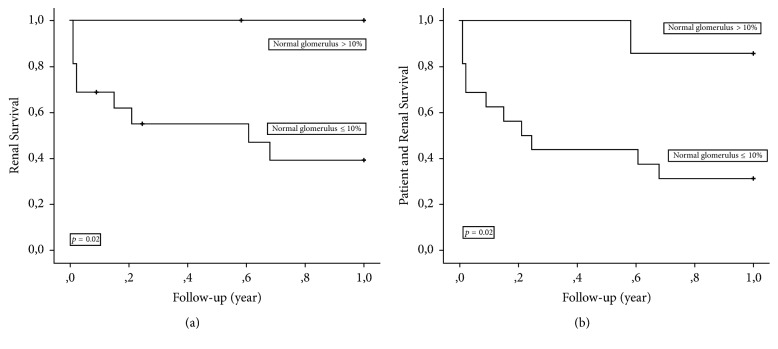
(a) Kaplan Meier plot demonstrating end-stage renal disease-free survival according to baseline percentage normal glomeruli ≤ versus >10%. (b) Kaplan Meier plot demonstrating end-stage renal disease-free patient survival according to baseline percentage normal glomeruli ≤ versus >10%.

Cumulative survival at 1 and 5 years without ESRD and ESRD/death in the study cohort and also after stratification for baseline eGFR and percentage of normal glomeruli is shown in Tables 2 and 3. The rate of 1- and 5-year cumulative survival without ESRD in the whole study cohort was 58% and 53%, respectively, whereas those rates after analyzing the combined end-point ESRD/death were 48% and 44%, respectively. Cumulative survival at 1- and 5-years without ESRD in patients with a baseline eGFR ≤15/>15 ml/min/1.73 m^2^ was 15%/85% (*p* = 0.002) and 15%/77% (*p* = 0.003), respectively. For the combined end-point ESRD/death event-free survival was 11%/71% (*p* = 0.004) and 11%/64% (*p* = 0.006), respectively. The corresponding numbers obtained after stratification of the study cohort according to the percentage of normal glomeruli ≤10%/>10% were 39%/100% (ESRD, *p* = 0.02) and 31%/86% (ESRD/death, *p* = 0.02) at 1-year, respectively. Five-year cumulative survival was 39%/83% (ESRD, *p* = 0.047) and 31%/71% (ESRD/death, *p* = 0.054), respectively. One-year ESRD and ESRD/death free survival in the study cohort stratified for eGFR ≤15 versus >15 ml/min/1.73 m^2^ and percentage normal glomeruli ≤10 versus >10% is shown in Figures 2(a)-2(b) and 3(a)-3(b).

## 4. Discussion

In the present study, we have shown that it is possible to risk stratify patients with ANCA-GN and a sclerotic histological presentation according to their risk of developing ESRD. We have found that ESRD-free long-term survival is particularly low in patients with sclerotic histology along with an eGFR ≤ 15 ml/min/1.73 m^2^. Meanwhile, patients with eGFR >15 ml/min/1.73 m^2^ at baseline had a much better long-term ESRD-free survival rate; their prognosis is approximately within the same range as that previously found for patients with a mixed or a crescentic histological pattern. We therefore suggest that an eGFR ≤ 15 ml/min/1.73 m^2^ represents the threshold for defining patients with sclerotic histology with a poor prognosis. The extent to which this group of patients needs less intensive immunosuppressive treatment requires further investigation and also depends on whether extrarenal inflammation is present.

In severe cases of ANCA-GN, the risk factors for ESRD and deaths are largely similar. Therefore, these events serve as competing end-points. Treating deaths as a censoring event can lead to underestimation of risk. To overcome this methodologic problem, we also analyzed cumulative survival without ESRD and death. With this end-point, 1-year survival fell from 58% to 48%. However, eGFR ≤15 ml/min/1.73 m^2^ continued to be a strong risk factor for this combined end-point. In cases with such a risk factor, 9 cases developed ESRD and 3 patients had died during the first year after being diagnosed with ANCA-GN. During the maintenance phase, the prognosis was improved, with only 1 of 11 patients progressing to ESRD and no deaths. Notably, this case of ESRD occurred in patients with an initial eGFR > 15 ml/min/1.73 m^2^; however, at this stage, only 1 patient with a baseline eGFR ≤ 15 ml/min/1.73 m^2^ remained in the study cohort.

In the present study, we also demonstrated that patients with ANCA-GN and sclerotic histology with ≤10% normal glomeruli, as indicated by biopsy, had a significantly lower ESRD-free survival rate. Further, cumulative ESRD-free survival was much higher with ≤10% normal glomeruli than with an eGFR of ≤15 ml/min/1.73 m^2^: 39% versus 15% at 1 year. Thus, in the present cohort, eGFR ≤15 ml/min/1.73 m^2^ was found to be the risk factor that best defines patients with a particularly poor prognosis.

Renal survival in our study cohort (i.e., 1-year: 58%) is approximately within the same range as has been reported by others. Chen et al. reported 1-year and 2-year ESRD-free survival rates in 36 patients with sclerotic ANCA-GN of 69% and 52%, respectively [15]. Berden et al. and Tanna et al. both reported a 1-year survival rate of 50% in the sclerotic group [14, 17]. Meanwhile, Chang et al. reported a substantially poorer outcome of 29% [30]. Estimated GFR and percentage normal glomeruli are well-documented risk factors for developing ESRD in patients with ANCA-GN in general [2, 3, 14]. However, to our knowledge, risk factor for progression have not yet been thoroughly analyzed, particularly in patients with sclerotic ANCA-GN. This is likely due to the small number of patients with sclerotic histology in earlier reports. Indeed, in a 2017 meta-analysis, Chen et al. performed a meta-analysis of 16 studies that reported the prognostic value of histologic classification [15] and found that the mean number of patients with sclerotic histology in these studies was 12.7 (range: 1–36). With the notable exception of the study by Chen et al., which included 36 patients, no other study has analyzed sclerotic histology in more than 17 patients.

Despite the robustness of our findings, there are some limitations to our study. First, the sample size is notably small, with only 23 patients having been included. However, due to a relatively high number of events, we were nevertheless able to demonstrate that an eGFR of ≤15 ml/min/1.73 m^2^ is a strong and highly statistically significant risk factor for progression to ESRD in patients with ANCA-GN and sclerotic histology. Future and preferably prospective studies are now necessary to confirm our findings and to define appropriate treatment strategies for this particular group of patents with ANCA-GN. Second, due to the nature of the NKBR as a kidney-focused registry, we could not report the clinical phenotype of ANCA-vasculitis (i.e., granulomatosis with polyangiitis, microscopic polyangiitis, or real limited vasculitis) or the extent of extrarenal disease activity, such as using the Birmingham vasculitis activity score. Third, although the general principles of treatment of ANCA-GN in Norway are known and follow standard international recommendations, we have no data regarding the treatment regimen used for specific patients in our study cohort [2, 12, 16]. Our observation that the outcomes of patients in our cohort are comparable to those in previous reports is indicative of the use of a standard treatment regimen.

## 5. Conclusions

We have demonstrated that prognosis in patients with ANCA-GN and sclerotic histology is variable and that risk stratification by baseline eGFR ≤15 ml/min/1.73 m^2^ versus >15 ml/min/1.73 m^2^ is clinically useful. Future prospective studies are necessary to define the appropriate treatment strategy for patients with sclerotic histology and baseline of eGFR ≤15 ml/min/1.73 m^2^.

## Figures and Tables

**Table 1:** Baseline characteristics in the total cohort and stratified for living patients without end-stage renal disease at the 1-year follow-up.

Characteristic	All, *n* = 23	Alive without ESRD 1-yr, *n* = 11	ESRD/death 1-yr, *n* = 12	*p*
Females (%)	13 (57%)	5 (45%)	8 (67%)	0.31
Mean age year (SD)	58 (21)	52 (21)	63 (21)	0.13
P-ANCA/MPO-ANCA (%)	18 (78%)	8 (73%)	10 (83%)	0.54
eGFR ml/min/1.73 m^2^ (SD)	36 (40)	52 (43)	21 (32)	0.007
Serum-albumin gram/liter (SD)	36 (10)	41 (11)	31 (5)	0.004
Systolic blood pressure mmHg (SD)	146 (26)	144 (28)	148 (24)	0.40
Diastolic blood pressure mmHg (SD)	80 (11)	80 (12)	79 (10)	0.65
Proteinuria gram/24 hours (SD)	2.2 (2.0)	3.4 (2.4)	2.0 (1.6)	0.95
Percentage normal glomeruli (SD)	9 (12)	15 (14)	4 (7)	0.02

ESRD: end-stage renal disease; yr: year; eGFR: estimated glomerular filtration rate.

**Table 2:** End-stage free survival at 1 and 5 years of follow-up in the total cohort and stratified according to baseline estimated glomerular filtration rate and percentage normal glomeruli, as indicated during biopsy.

Characteristic	*N*	ESRD	1-yr	5-yr	*p* value
All	23	10	58%	53%	
eGFR ≤ 15 ml/min/1.73	9	7	15%	15%	*p* = 0.003
eGFR > 15 ml/min/1.73	14	3	85%	77%	
Normal glomeruli ≤ 10%	16	9	39%	39%	*p* = 0.047
Normal glomeruli > 10%	7	1	100%	83%	

ESRD: end-stage renal disease; yr: year; eGFR: estimated glomerular filtration rate.

**Table 3:** End-stage free patient survival at 1 and 5 years of follow-up in total cohort and stratified according to baseline estimated glomerular filtration rate and percentage normal glomeruli, as indicated during biopsy.

Characteristic	*n*	ESRD/Death	1-yr	5-yr	*p* value
All	23	13	48%	44%	
eGFR ≤ 15 ml/min/1.73	9	8	11%	11%	*p* = 0.006
eGFR > 15 ml/min/1.73	14	5	71%	64%	
Normal glomeruli ≤ 10%	16	11	31%	31%	*p* = 0.054
Normal glomeruli > 10%	7	2	86%	71%	

ESRD: end-stage renal disease; yr: year; eGFR: estimated glomerular filtration rate.

## Data Availability

The corresponding author keeps an anonymized version of the research file.
